# The epidemiology and the pathogen distribution of pediatric dacryocystitis in Chinese population 2017–2022

**DOI:** 10.1186/s13052-024-01582-4

**Published:** 2024-01-22

**Authors:** Zhihong Sun, Mingchao Li, Huiqing Sun

**Affiliations:** 1 Department of Neonatology, Children’s Hospital Affiliated to Zhengzhou University, Henan Children’s Hospital, Zhengzhou Children’s Hospital, Henan, China; 2 Department of Neonatology, Shangqiu People’s Hospital, Henan, China

## Abstract

**Background:**

Dacryocystitis is a common disease in pediatric ophthalmology. Analysis of basic information, flora distribution, and characteristics of information on drug-resistant bacteria in children with dacryocystitis for 6 years, providing evidence for ophthalmologic infection prevention and clinical management strategies.

**Methods:**

A retrospective cohort study was conducted to evaluate the demographics of dacryocystitis in children and microbiological characteristics of secretion cultures, and to analyze the basic information, distribution of pathogenic bacteria, drug resistance, and to plot trendsand distribution pie charts according to the years.

**Results:**

This study recruited 5791 specimens. Decreased incidence of dacryocystitis from 2020 to 2022 (including the COVID-19 pandemic). The age of highest incidence of dacryocystitis is infancy, followed by the neonatal period, and the incidence decreased with age. *Streptococcus pneumoniae* had the highest percentage in 2017, and the overall trend was decreasing, the difference was statistically significant (*p* < 0.001); *Streptococcus mitis* showed an overall increasing trend, with the highest incidence in 2022 and the lowest in 2017, with a statistically significant difference (*p* < 0.001); *Haemophilus influenzae* was the most common gram-negative bacteria with an overall decreasing trend (*p* < 0.001); The incidence of Catamoeba and Stenotrophomonas varied from year to year, with statistically significant differences (*p* = 0.010, *p* = 0.033, respectively). Methicillin-resistant *Staphylococcus aureus* (MRSA) had the lowest incidence in 2017 and 2022 the highest incidence in 2022, with a statistically significant difference in incidence between years (*p* = 0.003); β-lactamase-positive was the most common type of resistance, and MRSA was the second, with statistically significant differences between years (*p* = 0.003, *p* < 0.001, respectively). *Streptococcus pneumoniae* is a common etiologic agent of dacryocystitis in all age groups.

**Conclusions:**

Dacryocystitis in children is significantly associated with age characteristics and infection-related pathogens, and infection prevention and control can help reduce the infection of related pathogens and the increase of new drug-resistant strains. Close monitoring of changes in pathogen distribution in ocular secretion cultures can help in early intervention and treatment of infectious dacryocystitis.

## Introduction

Dacryocystitis is the most common ophthalmic disease among children, especially in neonate. Persistence of Hasner’s membrane is the most common cause of dacryostenosis [1, 2], with more than 90% of cases showing perforation during the first 4 to 6 weeks [3]. If no perforation occurs, the tears gather in the lacrimal duct and sac until the system is full and therefore may become infected [4]. The clinical manifestation is tear discharge, accompanied by increased viscous or purulent drainage, which could lead to keratitis, acute dacryocystitis, lacrimal sac abscess, and other serious consequences if not treated in time [4, 5]. Congenital dacryocyst is a cyan cystic mass in the inner canthus of newborns, and is caused by obstruction at both ends of the diversion channel [6]. Some study reported that in children, nearly 60% of patients with congenital dacryocyst eventually develop a peri-dacryocyst infection [7, 8]. Dacryocystitis is mainly caused by Gram-positive cocci, especially *Streptococcus pneumoniae*; due to the misuse of anti-Gram-positive cocci antibiotics, Gram-negative Enterobacteriaceae have risen in the proportion of dacryocystitis pathogens [9]. However, there is no data about the epidemiology of pediatric dacryocystitis and the distribution of dacryocystitis pathogens. We have presented the epidemiology and pathogen distribution of dacryocystitis in children in Henan, China over the past 6 years, providing a basis for ophthalmic infection prevention and clinical management strategies.

## Methods

### Study design

The study was a retrospective cohort design including children with dacryocystitis and positive ocular secretion culture. Data were extracted from the electronic medical records of Children’s Hospital affiliated to Zhengzhou University from January 2017 to December 2022. Pathogens isolated from ocular secretions samples from the ophthalmology department were selected for the first time in each child, while duplicate strains from the same case and site were excluded. Preliminary 2017 prevalence of *Streptococcus pneumoniae* is 20.7%, with a projected decline of 1/3, and more than 499 cases need to be included in each group of this study.

### Subjects

The study subjects included children with dacryocystitis diagnosed and ocular secretion culture was positive. Dacryocystitis is diagnosed in children who meet both of the following criteria. (1) The child has tear overflow and/or increased secretions. (2) Mucopurulent or purulent secretions are seen when the lacrimal sac is compressed. (3) Lacrimal flushing in the absence of a swallowing maneuver reveals cloudy or purulent secretions overflowing from the upper lacrimal punctum. Exclusion criteria: increased or decreased tearing other than dacryocystic factors, history of maxillofacial or ocular surgery, history of antibiotic use 1 week prior to specimen sampling, cataracts, conjunctivitis, wheals, etc.

Their basic information, flora distribution, and characteristics of information on drug-resistant bacteria were analyzed. Such as, Extended Spectrum Beta-Lactamases (ESBLs), methicillin-resistant coagulase-negative staphylococci (MRCNS), methicillin-resistant *Staphylococcus aureus* (MRSA), carbapenem-resistant Enterobacteriaceae (CRE) carbapenem-resistant *Acinetobacter baumannii* (CRABA). All patients with tear epistemology due to lacrimal disease, a history of maxillofacial surgery, trauma, and tumor, and patients who received topical or systemic antibiotics within 1 week prior to microbial culture were excluded. This study was approved by the ethics committee of Children’s Hospital affiliated to Zhengzhou University.

### Definitions

Acute or chronic dacryocystitis is diagnosed based on the patient’s history, signs, and symptoms. Typical manifestations are redness and swelling in the lacrimal sac area, local tenderness, swollen lymph nodes in front of the ear, and even the formation of abscesses and lacrimal cyst fistulas [10].

Children are defined as under the age of 18. The maximum age of the subjects included in our study was 12 years, so there were no subgroups aged 12–18 years.

Neonate: from birth to 28 days, infancy: from 29 days after birth to 1 year old, toddlerhood: from 1 year old to before 3 years old, pre-school age: from 3 years old to before 6 years old, school age: from 6 years old to before 12 years old.

MDRB (multidrug-resistant bacteria) are bacteria that exhibit resistance to three or more classes of commonly used antimicrobial drugs to which they are usually susceptible at the same time, making anti-infective treatment extremely difficult. Common mechanisms of multi-drug resistant bacteria include: ESBLs: plasmid-mediated β-lactamases capable of hydrolyzing β-lactam antimicrobial drugs such as penicillins, cephalosporins and monocyclic amides. MRCNS, MRSA: Coagulase-negative staphylococci and *Staphylococcus aureus* resistant to most beta-lactams, antistaphylococcal penicillins (e.g., methicillin, benzoxiline), and cephalosporins. CRE, CRPAE: Enterobacteriaceae and *Pseudomonas aeruginosa* that are resistant to any of the carbapenem antibiotics, such as imipenem and meropenem, or are confirmed to produce carbapenemases.

### Sample collection and microbiologic culture

Specimen taken: first rinse the eye with normal saline to reduce bacterial contamination, then dry it with sterile cotton balls, then press and squeeze the lacrimal sac section, and swab the purulent secretions taken by sterile cotton swabs.

Specimens were cultured using a BD Bactectm FX400 Automated Blood Culture Analyzer (BD Corporation, USA), Columbia Blood Agar Base and Chocolate Blood Agar PlateMedium (Autubio Company, China), and identified using microflex™ (BRUKER Company, Germany).

### Data analysis

Statistical analysis of the data was performed using SPSS 26.0 (IBM Corporation, USA). The measurement data (non-normal distribution) were expressed as median(M) and 25th percentile, 75th percentile (p25, p75), and the K-W test was used for comparison between groups. The count data were expressed as rate (%), and the χ2 test was used for comparison between groups. The difference was considered statistically significant at *p* < 0.05. Image drawing using GraphPad Prism10 (GraphPad Software, USA) and Excel (Microsoft, USA).

## Results

This study has compared the number and proportion of different pathogens over 6 years from January 1, 2017, to December 31, 2022 (including period of Coronavirus disease-19 (COVID-19) pandemic, early 2020 to 2022) in our Hospital. A total of 5791 cases were included, including 3391 males and 2400 females.

### Baseline characteristics

**Fig. 1:**
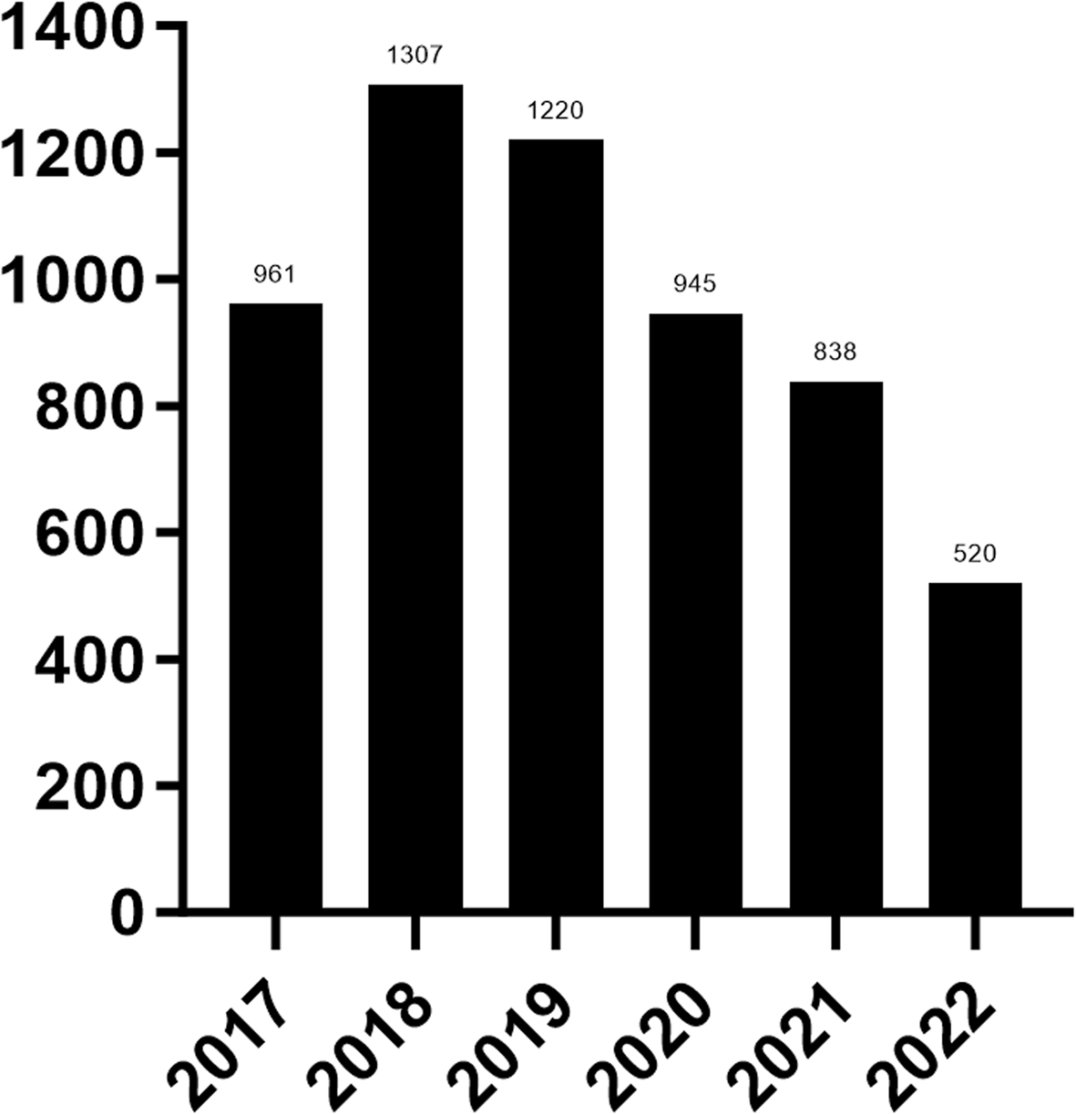
Number of positive cases of secretion culture in patients with dacryocystitis

Among all children between 2017 and 2022: the proportion of infancy was the highest in all years, followed by toddler hood, and the overall detection rate showed a downward trend in age. The proportion of multiple infections was highest in 2020 and lowest in 2017, with a statistically significant difference. In all specimens, the proportion of multiple infections was 13.6%. Higher rates of dacryocystitis in male children in all years. As shown in Table 1. The number of cases detected from 2020 to 2022 (COVID-19 pandemic) was lower than that from 2017 to 2019, but there was no statistically significant difference (*p* = 0.074), as shown in Fig. 1.

**Table 1:** Clinical data of positive secretion culture in children with dacryocystitis from 2017 to 2022

	2017	2018	2019	2020	2021	2022	Total
	(*n* = 961)	(*n* = 1307)	(*n* = 1220)	(*n* = 945)	(*n* = 838)	(*n* = 520)	(*n* = 5791)
Month-old, M (P25, P75)	4.27 (3.20,6.12)	4.67 (3.47,6.80)	4.53 (3.47,6.49)	5.13 (3.83,7.08)	5.35 (3.87,7.67)	5.53 (4.00,7.30)	4.87 (3.57,6.90)
Neonatal period, n (%)	30(3.12)	48(3.67)	33(2.7)	16(1.69)	8(0.95)	10(1.92)	145(2.50)
Infancy, n (%)	882(91.78)	1152(88.14)	1123(92.05)	859(90.90)	750(89.50)	473(90.96)	5239(90.47)
Toddler hood, n (%)	43(4.47)	99(7.57)	55(4.51)	68(7.20)	73(8.71)	35(6.73)	373(6.44)
Preschool age, n (%)	4(0.42)	7(0.54)	8(0.66)	2(0.21)	6(0.72)	0(0)	27(0.47)
School age, n(%)	2(0.21)	1(0.08)	1(0.08)	0(0)	1(0.12)	2(0.38)	7(0.12)
multiple infection, n(%)	64(6.7)	132(10.6)	195(16.0)	180(19.0)	136(16.2)	82(15.7)	789(13.6)
Males, n (%)	567(59.00)	755(57.77)	722(59.18)	544(57.57)	488(58.23)	315(60.58)	3391(58.56)

*M* median(M), *P25* 25th percentile, *P75* 75th percentile

**Fig. 2:**
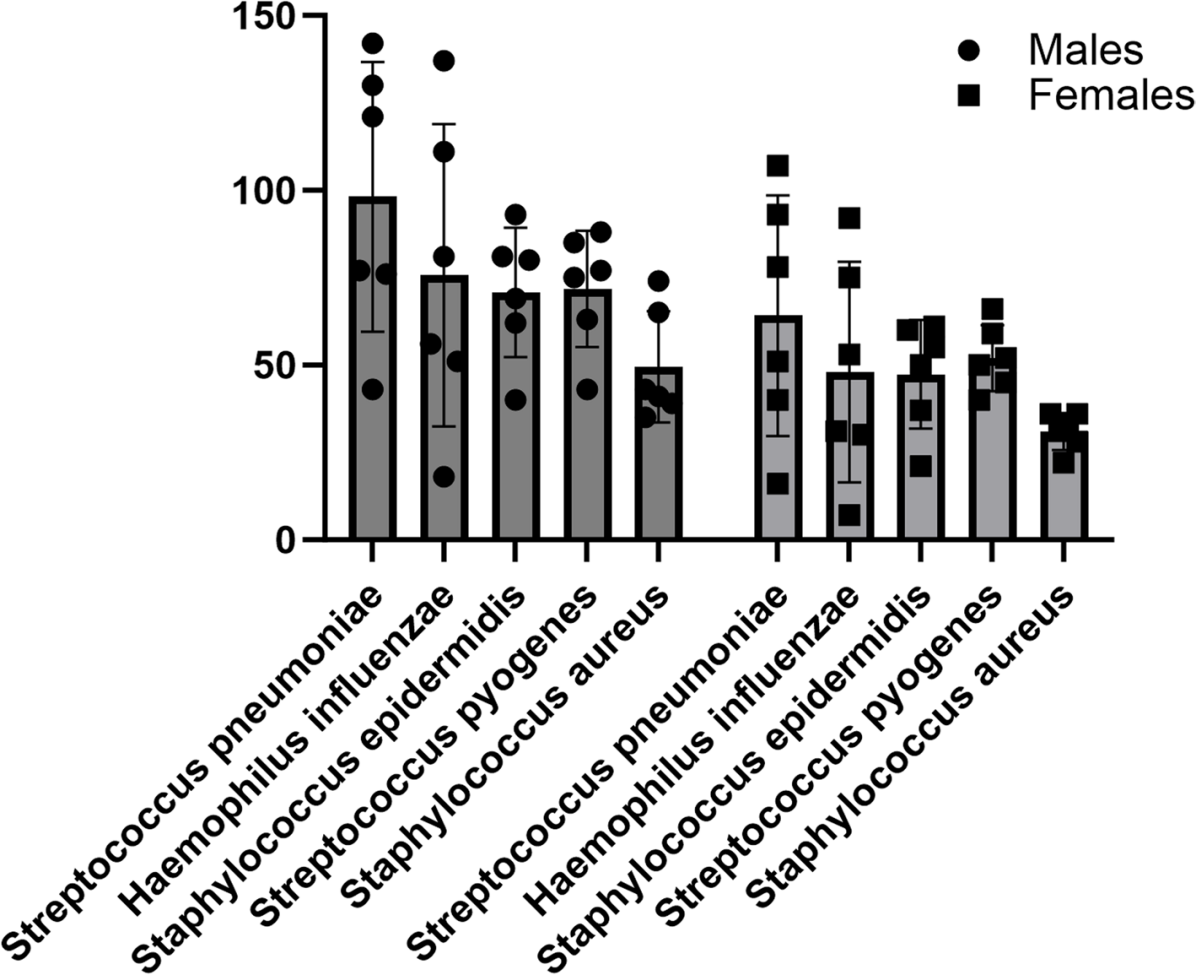
Characterization of the main pathogens in patients of different sexes

The aetiology of children with dacryocystitis in males is approximately the same as in girls. *Streptococcus pyogenes* is more common in females and is in second place. as shown in Fig. 2.

### Etiology changes with years

In this study, 3221 strains of Gram-positive bacteria, 2447 strains of Gram-negative bacteria, 123 strains of fungi.

Among the gram-positive bacteria, *Streptococcus pneumoniae* had the higher percentage in all years except 2020, but the overall trend was decreasing, with highest incidence in 2017 and lowest in 2022, the difference was statistically significant (*p* < 0.001); *Streptococcus mitis* showed an overall increasing trend, with the highest incidence in 2022 and the lowest in 2017, with a statistically significant difference (*p* < 0.001); Human Staphylococcus had the highest incidence in 2022 and the lowest in 2019, with a statistically significant difference (p < 0.001); and there was no statistically significant difference in the incidence of *Staphylococcus epidermidis* and *Staphylococcus aureus* among the years (*p* > 0.05).

Among the gram-negative bacteria, *Haemophilus influenzae* was the most common pathogen with an overall decreasing trend, with the highest incidence in 2018 and the lowest incidence in 2022, with a statistically significant difference (*p* < 0.001); Catamoeba had the highest incidence in 2018 and the lowest incidence in 2020, with a statistically significant difference (*p* = 0.010); *Stenotrophomonas maltophilia* had the 2019 had the highest incidence and the lowest incidence in 2020, with a statistically significant difference (*p* = 0.033); *Pseudomonas aeruginosa* and *Haemophilus parainfluenzae* did not have statistically significant differences in incidence across years (*p* > 0.05).

Among fungal infections: the highest incidence of pseudomonas albicans in 2017 and not detected in 2021 and 2022, with a statistically significant difference (*p* = 0.011); the highest incidence of Nearly Smooth Pseudomonas in 2018 and not detected in 2022, with a statistically significant difference (*p* = 0.012); the highest incidence of Nearly Smooth Candida in 2022 and not detected in 2017, with a The difference was statistically significant (*p* < 0.001). Shown in Table 2.

**Table 2:** The pathogen distribution of ocular secretions

	2017	2018	2019	2020	2021	2022	Χ^2^	*p* value
	(*n* = 961)	(*n* = 1307)	(*n* = 1220)	(*n* = 945)	(*n* = 838)	(*n* = 520)		
Gram-positive bacteria								
* Streptococcus pneumoniae*, n (%)	199(20.7)	249(19.1)	219(18.0)	117(12.4)	127(15.2)	59(11.3)	42.995	0.000
* Staphylococcus epidermidis*, n (%)	140(14.6)	154(11.8)	125(10.2)	124(13.1)	99(11.8)	61(11.7)	8.616	0.125
* Streptococcus mitis*, n (%)	103(10.7)	144(11.0)	153(12.5)	127(13.4)	127(15.2)	97(18.7)	27.444	0.000
* Staphylococcus aureus*, n (%)	77(8.0)	110(8.4)	95(7.8)	74(7.8)	67(8.0)	57(11.0)	5.421	0.367
Human Staphylococcus, n (%)	2(0.2)	5(0.4)	1(0.1)	14(1.5)	2(0.2)	10(1.9)	32.059	0.000
Gram-negative bacteria								
* Haemophilus influenzae*, n (%)	134(13.9)	229(17.5)	186(15.2)	81(8.6)	87(10.4)	25(4.8)	82.98	0.000
* Staphylococcus aureus*, n (%)	60(6.2)	106(8.1)	74(6.1)	42(4.4)	65(7.8)	31(6.0)	14.988	0.010
* Stenotrophomonas maltophilia*, n (%)	38(4.0)	37(2.8)	33(2.7)	48(5.1)	35(4.2)	23(4.4)	12.101	0.033
* Pseudomonas aeruginosa*, n (%)	29(3.0)	28(2.1)	34(2.8)	33(3.5)	24(2.9)	13(2.5)	4.113	0.533
* Haemophilus parainfluenzae*, n (%)	23(2.4)	31(2.4)	35(2.9)	18(1.9)	18(2.1)	4(0.8)	8.657	0.124
Fungi								
Pseudomonas albicans, n (%)	8(0.8)	5(0.4)	2(0.2)	1(0.1)	0(0.0)	0(0.0)	12.299	0.011
Nearly Smooth Pseudomonas, n (%)	8(0.8)	18(1.4)	9(0.7)	3(0.3)	4(0.5)	0(0.0)	13.934	0.012
Nearly Smooth Candida, n (%)	0(0.0)	2(0.2)	5(0.4)	10(1.1)	6(0.7)	8(1.5)	23.662	0.000

**Fig. 3:**
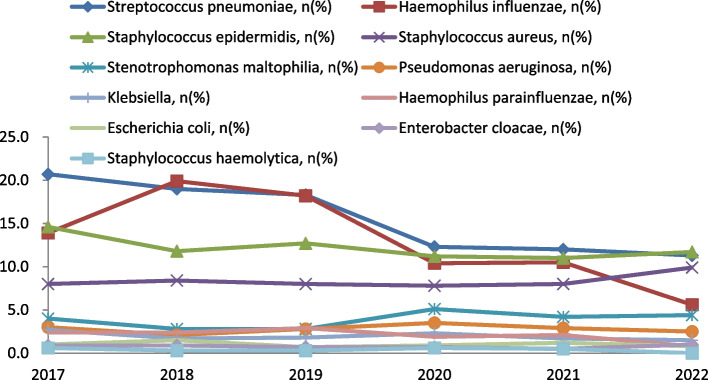
Changes in the major flora of culture-positive ocular secretions in patients with dacryocystitis, 2017 to 2022

*Streptococcus pneumoniae*, *Haemophilus influenzae*, and Pseudomonas albicans decreased in incidence during the COVID-19 pandemic; Streptococcus bradypneumoniae, *Stenotrophomonas maltophilia*, and Candida proximalis increased during the COVID-19 pandemic, and there was no significant change in the detection rate of *Staphylococcus epidermidis*, *Staphylococcus aureus*, *Pseudomonas aeruginosa*, Klebsiella spp., *Haemophilus parainfluenzae*, *Escherichia coli*, and *Enterobacter cloacae*, staphylococcus hemolyticus. Shown in Fig. 3.

### Drug-resistant bacteria analysis

In the analysis of multidrug-resistant pathogens for dacryocystitis, ESBLs had the highest incidence in 2020 and were not detected in 2022, with no statistically significant difference between years (*p* = 0.211); MRSCN had the highest incidence in 2017 and were not detected in 2020, with no statistically significant difference in incidence between years (*p* = 0.736); MRSA had the lowest incidence in 2017 and 2022 the highest incidence in 2022, with a statistically significant difference in incidence between years (*p* = 0.003); CRE had the highest incidence in 2020, and was not detected in 2017, 2019, and 2022, with no statistically significant difference between years (*p* = 0.415); and CRPAE had the highest incidence in 2020, and was not detected in 2017 and 2021, with no statistically significant (*p* = 0.246); β-lactamase-positive bacteria had the highest incidence in 2017 and the lowest incidence in 2022, and the difference in incidence between years was statistically significant (*p* < 0.000). Show in Table 3.

**Table 3:** Culture of drug-resistant bacteria in ocular secretions from patients with dacryocystitis, 2017 to 2022

	2017	2018	2019	2020	2021	2022	Χ^2^	*p* value
	(*n* = 961)	(*n* = 1307)	(*n* = 1220)	(*n* = 945)	(*n* = 838)	(*n* = 520)		
ESBLs, n(%)	2(0.21)	7(0.54)	2(0.16)	6(0.63)	4(0.48)	0(0)	6.664	0.211
MRCNS, n(%)	2(0.21)	1(0.08)	2(0.16)	0(0)	1(0.12)	1(0.19)	2.882	0.736
MRSA, n(%)	8(0.83)	14(1.07)	12(0.98)	13(1.38)	19(2.27)	15(2.88)	17,688	0.003
CRE, n(%)	0(0)	1(0.08)	0(0)	2(0.21)	1(0.12)	0(0)	4.006	0.415
CRPAE, n(%)	0(0)	1(0.08)	1(0.08)	3(0.32)	0(0)	1(0.19)	5.071	0.246
β-lactamase positive, n(%)	71(7.39)	93(7.12)	54(4.43)	33(3.49)	35(4.18)	7(1.35)	44.489	0.000

*ESBLs* ultra broad-spectrum β-lactamase-producing bacteria, *MRCNS* methicillin-resistant coagulase-negative staphylococci, *MRSA* methicillin-resistant *Staphylococcus aureus*, *CRE* carbapenem-resistant Enterobacteriaceae, *CRABA* carbapenem-resistant *Acinetobacter baumannii*

### Etiology changes with age

**Fig. 4:**
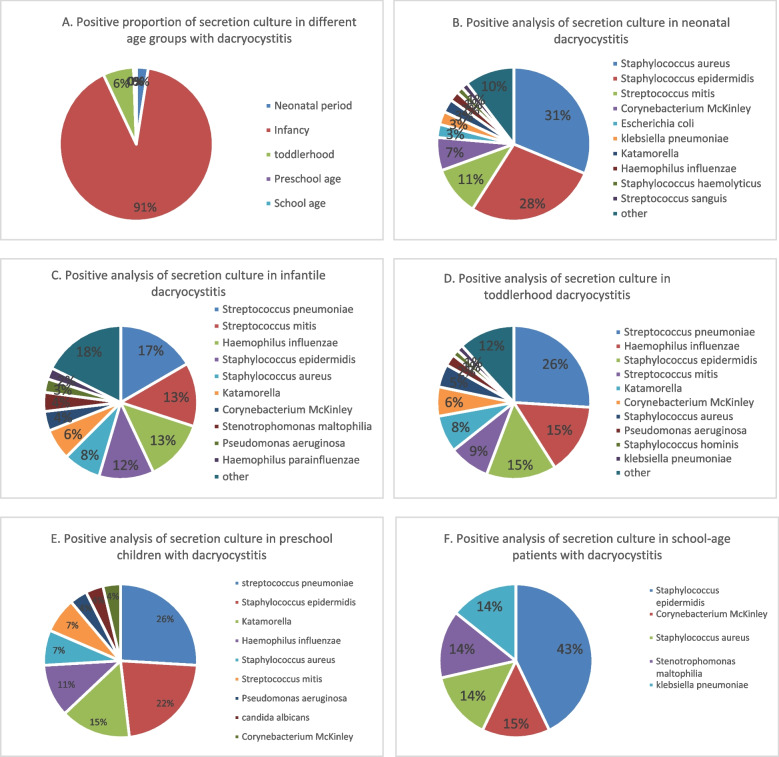
Percentage of culture-positive dacryocystitis and major flora in various age groups

Analyzing the pathogenic characteristics of each age group, infancy accounted for the majority of patients in 91% of all cases with positive ocular secretion cultures. This was followed by early childhood and neonatal period. The incidence of positive culture of dacryocystitis secretions decreased significantly with increasing age. The most common infections in neonatal period were *Staphylococcus aureus*, *Staphylococcus epidermidis*, and Streptococcus bradypneumoniae. The most common infections in infancy were, in order, *Streptococcus pneumoniae*, Streptococcus bradypneumoniae, and *Haemophilus influenzae*. The most common infections in early childhood are, in order, *Streptococcus pneumoniae*, *Haemophilus influenzae*, and *Staphylococcus epidermidis*. The most common infections in the preschool years were *Streptococcus pneumoniae*, *Staphylococcus epidermidis*, and Catamorium. The most common infections during the school years were *Staphylococcus epidermidis*. Show in Fig. 4.

## Discussion

The data in this paper show that the incidence of dacryocystitis varies from year to year, and the distribution of pathogens in purulent drainage cultures from children’s eyes also varies. Among the 6 years of data analyzed in this paper, 3 years of the COVID-19 pandemic in China were included. As the largest public health event in recent years, this may provide a reference for pathogen changes in subsequent similar events (widespread respiratory epidemics or other events that require reduced human mobility). We need to continuously monitor the epidemiologic changes of various pathogens in the ophthalmic system and their impressionable factors.

The average age of children with dacryocystitis rose for the past few years, but the median age remained that of infancy. In a study by Daphna Prat et al. [11]. It was found that out of the 169 cases studied by them, 55% of the children were below 3 months of age, the mean age was 1.6 ± 2.9 years. Infancy and the neonatal period are the main groups in which dacryocystitis occurs in children and may be associated with the following factors: Congenital disorders of tear duct development are the main causes of dacryocystitis, including unruptured Hasner’s valves, blockage of the tear ducts or conjunctivitis due to obstetrical infections, or stenosis of the tear ducts at the bones [12]. Children in infancy and the neonatal period are more likely to become infected by touching their hands to the eye when exposed to their surroundings without good hygiene.

The male to female ratio of children with dacryocystitis was approximately 3:2 in all years, suggesting that the male to female incidence of dacryocystitis does not vary significantly and that events like the COVID-19 pandemic do not affect the gender of the incidence. And in Ali’s study, dacryocystitis have shown a strong female preponderance [13]. However, in Ban Luo’s study, although the adult population was predominantly female (71.9%), males were more prevalent among children (59.0%) [14]. Obstruction of the nasolacrimal duct in children is a major influence on the development of dacryocystitis, and in Bruce M Schnall’s study [15], dilation of the lacrimal sac due to valvular anomalies was more common in female children, and may be resolved in the obstetrics department. This may be a reason why there were more cases of dacryocystitis in boys in this study. The rate of multiple infections in this study was 13.6%, which is comparable to previous rates of polymicrobial infections (7–30%) [14]. The odds of multiple infections have had a significant impact in recent years.

The most common of the COVID-19 co-infections are *Streptococcus pneumoniae*, followed by *Klebsiella pneumoniae* and *Haemophilus influenzae* [16]. Nevertheless, in cultures of ocular secretions from children with dacryocystitis, the proportion of *Streptococcus pneumoniae* and *Haemophilus influenzae* decreased compared to before. Consistent and significant sustained reductions in invasive disease caused by *Streptococcus pneumoniae*, *Haemophilus influenzae* and *Neisseria meningitidis* have been observed in every country that has adopted new crown pneumonia control measures [17]. Analyzing *Streptococcus pneumoniae* and *Haemophilus influenzae* commonality: they are both commensals of the human nasopharynx, are co-located within the biofilm to ensure that they are all persistent, and therefore often show the same tendency during the course of an epidemic [18]. Reduced transmission of new coronaviruses as a result of their introduction of respiratory management, restrictions on the movement of people and other measures, which may help to explain their downward trend. *Streptococcus mitis* belongs to the same genus Streptococcus as *Streptococcus pneumoniae*, and its and *Streptococcus pneumoniae* proteins cross-react [19]. Fungal infections account for a minority of dacryocystitis pathogens, but there has been an upward trend in recent smooth Candida infections since the COVID-19 pandemic, and fungal infections should be considered in the setting of suboptimal treatment. This study suggests that the COVID pandemic influences changes in the infectious flora of dacryocystitis in children.

Multidrug-resistant bacteria (MDRB) [20] refers to bacteria that are resistant to three or more commonly used antimicrobial drugs that are usually sensitive, making anti-infective treatment much more difficult. Multidrug-resistant bacteria in dacryocystitis are relatively rare in this region. The most common type of resistance is β-lactamase positive, catabolizing penicillin, cephalosporin, etc. Using other types of antibiotics or adding drugs containing enzyme inhibitors may have better therapeutic effects. MRSA also accounted for the major portion of drug-resistant bacteria,which proportion of MASA increased year by year. In most studies, *Staphylococcus aureus* is prevalent in young children, especially in the neonatal period and infancy [21]. The overall per-pathogen analysis shows significant increases in infections by MRSA [22], although this study was of blood specimen cultures, it suggests that MRSA is increasing in addition to bloodstream infections. In this paper, year-to-year changes in *S. aureus* were not statistically different, but the increased rate of MRSA infections may be related to the rapid spread of *Staphylococcus aureus* carrying pathogenic genes in the environment [23]. Effective antibiotic application and a reasonable course of treatment can help reduce the spread of drug-resistant bacteria.

Infants make up the majority of all patients with dacryocystitis, 91%. The common flora of positive secretion cultures were analyzed in all age groups: *Streptococcus pneumoniae* was consistently well represented in all age subgroups and was the most common culture-positive organism from 1 month to 6 years of age. The percentage of gram-negative bacteria such as *Haemophilus influenzae* and Cattamoeba was also high, consistent with the rising trend of gram-negative infections in neonates. Dacryocystitis is mainly caused by gram-positive bacteria, the proportion of gram-negative bacteria is on the rise due to the use of antibiotics [3, 14]. Although Gram-positive bacteria are still predominant, some rare Gram-negative bacteria have become increasingly common in dacryocystitis in recent years. Sulfonamide and quinolone antibiotics can be applied empirically for the pathogenic characteristics of each age group [24]. A proportion of children can be cured by massage plus antibiotic eye drop treatment, and if this is not possible, tear duct exploratory surgery can be performed in due course [25].

Generally speaking, dacryocystitis is treated empirically in outpatient settings without the need for cultivation. Obtaining secretion culture data and selecting appropriate antibiotics is an effective method for treating dacryocystitis, and can prevent delayed antibiotic replacement when facing drug-resistant microorganisms [14]. In addition, the inclusion of the 3 years affected by COVID-19 in the data studied herein informs the prediction of changes in the pathogen of dacryocystitis when similar public health events recur.

## Conclusion

In conclusion, from January 1, 2017, to December 31, 2022, *Streptococcus pneumoniae* and *Haemophilus influenzae* are the most common Gram-positive and Gram-negative organisms in all age group there is a decreasing trend. However, *Streptococcus mitis* and *Stenotrophomonas maltophilia* have seen an increase in their incidence in recent years. During in the period of COVID - 19 pandemic, the number of cases of dacryocystitis in children decreased as the time passed, and the distribution of pathogens changed. For MDR, β - lactamase-positive bactexria are the most common type of drug resistance, followed by MRSA. And the incidence of MRSA is on the rise. Close monitoring of changes in pathogen distribution in ocular secretion cultures can help in early intervention and treatment of infectious dacryocystitis.

## Data Availability

The datasets used and/or analyzed during the current study are included in this published article.

## Abbreviations

COVID-19: Coronavirus disease-19
MDRB: Multidrug-resistant bacteria;
ESBLs: Extended Spectrum Beta-Lactamases
MRCNS: Methicillin-resistant coagulase-negative staphylococci
MRSA: Methicillin-resistant *Staphylococcus aureus*
CRE: Carbapenem-resistant Enterobacteriaceae
CRABA: Carbapenem-resistant *Acinetobacter baumannii*
